# Utility of Tofacitinib in Steroid-Refractory Acute Severe Ulcerative Colitis

**DOI:** 10.7759/cureus.71485

**Published:** 2024-10-14

**Authors:** Mukesh K Ranjan, Pradeep Neupane, Bigyan Maharjan

**Affiliations:** 1 Gastroenterology and Hepatology, Chitwan Medical College and Teaching Hospital, Bharatpur, NPL

**Keywords:** acute severe colitis, acute severe ulcerative colitis, fulminant colitis, janus kinase inhibitors, rescue therapy, steroid refractory, tofacitinib

## Abstract

In recent years, tofacitinib has been used in patients with acute severe ulcerative colitis (ASUC) as a rescue therapy with encouraging success rates. We present details of four patients with steroid-refractory ASUC treated with tofacitinib. All the patients were biologics-naive. Tofacitinib was initiated in a dose of 10 mg three times daily in three patients and 10 mg twice daily in the remaining patient. Three of the four patients improved and were discharged in clinical remission. These patients continue to be in colectomy-free and steroid-free remission on follow-up. The remaining patient did not respond to tofacitinib and required a colectomy. No adverse event related to tofacitinib use was noted in any of these four patients.

## Introduction

Acute severe ulcerative colitis (ASUC) is the most severe form of ulcerative colitis (UC). It is an emergency condition requiring inpatient management. About 20-30% of the patients with UC develop ASUC at some point during the disease course, and about one-third of the patients with UC have ASUC as the index presentation [1]. Corticosteroids have been the mainstay of treatment ever since the identification of their role in ASUC patients in 1954 by Truelove and Witts. It was seen that mortality significantly improved with the use of steroids (24% in the placebo group vs. 7% in the corticosteroid group, p=0.02) [2]. However, about 30-40% of patients with ASUC fail to respond to steroids. These patients require rescue therapy, which could be either medical or surgical in the form of a colectomy. Ciclosporin and infliximab are the best-known and widely used medical rescue therapies. A short-term response of 70-80% established both of these agents as rescue therapy of choice [3]. Both ciclosporin and infliximab have comparable efficacies in preventing colectomy [4,5]. However, despite an increase in the use of ciclosporin and infliximab as rescue therapy, a further decrease in colectomy rate is not seen [6]. Management of ASUC is a time-bound exercise with predefined checkpoints to assess the response to the pharmacologic therapy. Patients who do not exhibit a satisfactory response to corticosteroids should be subjected to rescue therapy on day three to five, and the response should be assessed on day five to seven. It is important to identify those patients who fail to respond to medical therapy and subject them to timely colectomy. It is seen that delaying colectomy beyond five to seven days results in higher mortality and postoperative complications. With the use of steroids, rescue therapy, and timely colectomy, the mortality rate has significantly reduced from 70% before the 1950s to 1-3% in the current time [7]. However, a considerable proportion of patients still fail to respond to steroid and standard second-line rescue therapy requiring colectomy.

In recent years, tofacitinib has been used as rescue therapy in ASUC with encouraging results [3]. Rapid onset of action, oral administration, and easy affordability make tofacitinib an excellent option [8]. Moreover, for low-middle-income countries where most patients cannot afford infliximab, tofacitinib can be the first choice of rescue therapy after steroid failure. Through this study, we aim to assess the effectiveness of tofacitinib in preventing colectomy in patients with steroid-refractory ASUC.

## Case presentation

This study was conducted in the Department of Gastroenterology and Hepatology at Chitwan Medical College and Teaching Hospital, Nepal, after obtaining ethical approval. We report the details of four cases of ASUC treated with tofacitinib between January 2022 and September 2024. The diagnosis of ASUC was made based on Truelove and Witts criteria [2]. The median age of the patients was 31.5 (22-37) years and included two males and two females. Three patients had left-sided colitis, and one had extensive colitis. All the patients were biologics and small-molecule naïve. Of the four patients, three were newly diagnosed cases of ulcerative colitis referred to our center and were diagnosed to have ASUC (Table 1). All the patients were managed as per the standard of care protocol, including the initiation of low molecular weight heparin. Baseline flexible sigmoidoscopy and colonic biopsies were obtained for cytomegalovirus (CMV) colitis. Stool cultures for pathogenic organisms, viral serology for hepatitis B virus (HBV), hepatitis C virus (HCV), human immunodeficiency virus (HIV), and chest X-rays were performed in all four patients at baseline. 

**Table 1:** Baseline disease characteristics of the patients. ASUC: acute severe ulcerative colitis; E1: proctitis, E2: left-sided colitis, E3: pancolitis (extensive colitis), EIM: extraintestinal manifestations

Patient	Age/Sex	Disease duration	Extent	EIM	Prior steroid course	Prior immunomodulator exposure	Prior biologic exposure	Prior ASUC	Comorbidities
1	22/M	6 weeks	E2	None	None	None	None	1^st^ episode	None
2	37/M	4 weeks	E3	None	None	None	None	1^st^ episode	None
3	30/F	4 weeks	E2	None	None	None	None	1^st^ episode	None
4	33/F	4 years	E2	None	Multiple	Azathioprine	None	3^rd^ episode	None

Baseline hemoglobin (g/dL), C-reactive protein (CRP) (mg/L), albumin (gm/dL), and ulcerative colitis endoscopic index of severity (UCEIS) were 11±1.18, 121.2±76.8, 2.32±0.49, and 4.75±0.5, respectively. All these patients were treated with hydrocortisone 100 mg six hourly at presentation. The response to steroids was assessed on day three by using Oxford criteria [9]. Tofacitinib was initiated on day three after the failure of steroids in three patients. The remaining patient received tofacitinib on day seven as a second rescue therapy after the failure of infliximab (5 mg/kg, single dose). The induction dose for tofacitinib was 10 mg twice daily in the first patient and 10 mg three times daily for nine doses, followed by 10 mg twice daily in the subsequent three cases (Table 2). Three patients (first, second, and fourth) improved and were discharged on tofacitinib alone in clinical remission defined by simple clinical colitis activity index (SCCAI) ≤2 with no blood in the stool. None of these patients received 5-aminosalicylic acids (5-ASA) for maintenance therapy. The third patient, a 30-year-old lady, however, did not respond to tofacitinib. There was a progressive worsening of general well-being and a significant drop in hemoglobin without any improvement in the frequency of bloody stool. The patient was subjected to a subtotal colectomy on day seven. She is awaiting second-stage surgery and is doing well on follow-up. There were no short-term adverse events noted with tofacitinib in any of these patients. Two of the patients (the first two) are in steroid and colectomy-free clinical remission on follow-up. There were no adverse events in these two patients till the last follow-up. 

**Table 2:** Outcome of tofacitinib as rescue therapy with baseline prognostic parameters. Hb: hemoglobin; CRP: C-reactive protein; Alb: albumin; UCEIS: ulcerative colitis endoscopic index of severity; BD: twice daily; TDS: thrice daily

Patient	Baseline parameters				Tofacitinib		Outcome	3- and 9- month steroid and colectomy-free survival	Total follow-up (months)	Complication
	Hb (g/dL)	CRP (mg/L)	Alb (g/dL)	UCEIS	Initiation dose	Position				
1	11.7	44.92	2.5	5	10 mg BD	1^st^ rescue therapy	Response	Yes	16	None
2	11.9	216	2.7	4	10 mg TDS for 3 days f/b 10 mg BD	2^nd^ rescue therapy	Response	Yes	9	None
3	11.1	149	1.6	5	10 mg TDS for 3 days	1^st^ rescue therapy	Colectomy	No	7	None
4	9.3	75	2.5	5	10 mg TDS for 5 days f/b 10 mg BD	1^st^ rescue therapy	Response	In remission on follow-up	1	None

## Discussion

In this case series of four patients, we demonstrate that tofacitinib is effective as rescue therapy in three-fourths of the steroid-refractory ASUC patients. Tofacitinib was effective as the first-line rescue therapy in two out of three patients. No adverse events were noted in any of the four patients treated with tofacitinib.

About 30-40% of patients with ASUC do not respond to steroids and require rescue therapy with infliximab or ciclosporin. Ciclosporin, due to its safety profile, remains the second choice after infliximab in real-world settings. However, it is important to acknowledge the fact that infliximab is not easily available to and affordable for most of the patients in developing nations [10,11]. Tofacitinib has been approved for moderate to severely active ulcerative colitis. It is an easily available, orally administered, affordable small molecule with onset of action as early as three days of initiation [12]. In recent years, tofacitinib has been used in ASUC as an effective rescue therapy. The first evidence of the utility of tofacitinib in steroid-refractory ASUC was reported by Berinstein et al. in 2019. Two of the four patients receiving tofacitinib avoided colectomy. Similar to our study, the study used tofacitinib as first-line rescue therapy in two patients [13]. In another case series where tofacitinib was used as the first-line rescue therapy after failure of the steroid, all four patients responded to the treatment [14]. In by far the largest multicenter study, which included 55 patients with prior exposure to either infliximab or ciclosporin, 40 (72.72%) patients responded to tofacitinib and avoided colectomy [15]. In another large case-controlled study including 40 patients with prior exposure to biologics, 34 (85%) patients improved with tofacitinib [16]. The same study observed that an initiation dose of 10 mg thrice daily, as opposed to 10 mg twice daily dosing, provided protection against colectomy. A very recent network meta-analysis, which included 21 studies with 2004 patients, evaluated the short-term colectomy-free survival with different rescue therapies in steroid-refractory patients with ASUC. The study found that tofacitinib had the best short-term colectomy-free survival as a rescue therapy (OR: 0.09; 95% CI: 0.02-0.52) compared to accelerated infliximab (OR: 0.16; 95% CI: 0.03-0.94), infliximab (OR: 0.2; 95% CI: 0.07-0.58), and tacrolimus (OR: 0.24; 95% CI: 0.06-0.96) [17]. Interestingly, the study found that ciclosporin was no better than placebo in reducing short-term colectomy.

There have been concerns about the safety profile of tofacitinib. In a post hoc analysis of 12,410 patients treated with tofacitinib for rheumatoid arthritis, psoriatic arthritis, and psoriasis, a heightened risk of thromboembolic phenomenon was observed specifically in patients ≥ 50 years of age and ≥1 cardiovascular risk factor. A caution with a 10 mg twice daily dose is suggested in patients with a high risk of thromboembolism [18]. However, the risk of venous thromboembolism in patients with ASUC treated with tofacitinib appears to be less. In a systematic review that included 134 patients with ASUC treated with tofacitinib, venous thromboembolic phenomena were reported in only one (0.7%) case. This in part may be due to routine use of low molecular heparin as prophylaxis and a younger patient population with ASUC. The commonest adverse events reported in this study were infections in 16 (11.9%) patients, followed by hair fall, nausea and vomiting, and skin rash each in one (0.7%) patient [19]. In our cohort of patients, no adverse events were noted in the short term (in all four patients) and in the long term (in two patients). In an updated systematic review article including 148 patients treated with tofacitinib, only seven (4.7%) of the patients had to stop tofacitinib due to adverse events, indicating that tofacitinib is tolerated well by the patients with ASUC [20].

## Conclusions

In conclusion, tofacitinib is an effective and safe therapy for preventing short-term colectomy in patients with steroid-refractory ASUC. This real-world data indicates that tofacitinib can be a valid option in low-middle-income countries where infliximab is not available and affordable by many. However, prospective studies with larger sample sizes are needed to validate the findings of this study. 

## References

[1] Bitton A, Buie D, Enns R (2012). Treatment of hospitalized adult patients with severe ulcerative colitis: Toronto consensus statements. Am J Gastroenterol.

[2] TR SC, WI LJ (1954). Cortisone in ulcerative colitis; preliminary report on a therapeutic trial. Br Med J.

[3] Gisbert JP, García MJ, Chaparro M (2023). Rescue therapies for steroid-refractory acute severe ulcerative colitis: a review. J Crohns Colitis.

[4] Laharie D, Bourreille A, Branche J (2012). Ciclosporin versus infliximab in patients with severe ulcerative colitis refractory to intravenous steroids: a parallel, open-label randomised controlled trial. Lancet.

[5] Williams JG, Alam MF, Alrubaiy L (2016). Infliximab versus ciclosporin for steroid-resistant acute severe ulcerative colitis (CONSTRUCT): a mixed methods, open-label, pragmatic randomised trial. Lancet Gastroenterol Hepatol.

[6] Turner D, Walsh CM, Steinhart AH, Griffiths AM (2007). Response to corticosteroids in severe ulcerative colitis: a systematic review of the literature and a meta-regression. Clin Gastroenterol Hepatol.

[7] Dong C, Metzger M, Holsbø E, Perduca V, Carbonnel F (2020). Systematic review with meta-analysis: mortality in acute severe ulcerative colitis. Aliment Pharmacol Ther.

[8] Dowty ME, Lin J, Ryder TF (2014). The pharmacokinetics, metabolism, and clearance mechanisms of tofacitinib, a janus kinase inhibitor, in humans. Drug Metab Dispos.

[9] Travis SP, Farrant JM, Ricketts C, Nolan DJ, Mortensen NM, Kettlewell MG, Jewell DP (1996). Predicting outcome in severe ulcerative colitis. Gut.

[10] Ranjan MK, Kumar P, Vuyyuru SK (2024). Thiopurines have sustained long-term effectiveness in patients with inflammatory bowel disease, which is independent of disease duration at initiation: a propensity score matched analysis. J Crohns Colitis.

[11] Makharia GK, Ramakrishna BS, Abraham P (2012). Survey of inflammatory bowel diseases in India. Indian J Gastroenterol.

[12] Hanauer S, Panaccione R, Danese S (2019). Tofacitinib induction therapy reduces symptoms within 3 days for patients with ulcerative colitis. Clin Gastroenterol Hepatol.

[13] Berinstein JA, Steiner CA, Regal RE (2019). Efficacy of induction therapy with high-intensity tofacitinib in 4 patients with acute severe ulcerative colitis. Clin Gastroenterol Hepatol.

[14] Kotwani P, Terdiman J, Lewin S (2020). Tofacitinib for rescue therapy in acute severe ulcerative colitis: a real-world experience. J Crohns Colitis.

[15] Uzzan M, Bresteau C, Laharie D (2021). Tofacitinib as salvage therapy for 55 patients hospitalised with refractory severe ulcerative colitis: A GETAID cohort. Aliment Pharmacol Ther.

[16] Berinstein JA, Sheehan JL, Dias M (2021). Tofacitinib for biologic-experienced hospitalized patients with acute severe ulcerative colitis: a retrospective case-control study. Clin Gastroenterol Hepatol.

[17] Huang CW, Yen HH, Chen YY (2024). Rescue therapies for steroid-refractory acute severe ulcerative colitis: a systemic review and network meta-analysis. J Crohns Colitis.

[18] Mease P, Charles-Schoeman C, Cohen S (2020). Incidence of venous and arterial thromboembolic events reported in the tofacitinib rheumatoid arthritis, psoriasis and psoriatic arthritis development programmes and from real-world data. Ann Rheum Dis.

[19] Mpakogiannis K, Fousekis FS, Christodoulou DK, Katsanos KH, Narula N (2023). The current role of Tofacitinib in acute severe ulcerative colitis in adult patients: A systematic review. Digestive and Liver Disease.

[20] Steenholdt C, Dige Ovesen P, Brynskov J, Seidelin JB (2023). Tofacitinib for acute severe ulcerative colitis: a systematic review. J Crohns Colitis.

